# The Influence of Self-Relevance and Cultural Values on Moral Orientation

**DOI:** 10.3389/fpsyg.2019.00292

**Published:** 2019-02-28

**Authors:** Junfeng Bian, Liang Li, Jianzhou Sun, Jie Deng, Qianwei Li, Xiaoli Zhang, Liangshi Yan

**Affiliations:** ^1^Centre for Mental Health Education, Changsha University of Science and Technology, Changsha, China; ^2^Hunan Normal University, Changsha, China; ^3^Department of Psychology, Centre for Research of Cultural Psychology and Behavior, Hunan Normal University, Changsha, China

**Keywords:** moral orientation, justice, care, degree of self-reference, differential effect, cultural value

## Abstract

Moral orientation refers to moral values that have a consistent guiding orientation toward an individual's moral cognition and behavior. Gilligan (1982) proposed that individuals have two moral orientations, namely “justice” and “care.” In the current study, we investigated the influence of self-relevance and cultural values on justice and care by using Single Attribute Implicit Association Test (SA-IAT). In Experiments 1 and 2, we adopted cultural icon prime paradigm to examine the effects of different self-referential stimuli (self, friend, and stranger) on implicit moral justice and care orientation under two cultural value conditions: traditionality, modernity, and neutral cultural values. Participants exhibited more difference toward different self-referential stimuli in the traditionality condition than in the modernity condition; the priming of traditional culture aggravated the differential order, whereas the priming of modernity weakened the differential order regarding implicitly just moral orientation. In the implicit care orientation, participants in the modern culture group exhibited the least difference to different self-referential stimuli compared with the other two groups, and the traditional group and the control group did not differ significantly. These findings indicate that psychological modernity weakens the degree of self-related effect in implicit justice and care orientation, whereas traditional culture aggravates the differential order in justice orientation. The current studies provide empirical support for theories relating moral orientation, also informing the literature on the role of self-relevance information and cultural values in moral decision making.

## Introduction

Moral orientation refers to moral values that have a consistent guiding orientation toward an individual's moral cognition and behavior and consists of two types: reward good cops and punish bad ones. Kyte (1996) suggested that moral orientation plays an integral role in moral decision-making.

Piaget and Kohlberg are recognized as representatives of the “justice” moral orientation. Kohlberg's (1976) proposed model for moral justice orientation focuses on adjudicating between individual interests or rights while solving moral dilemmas. This orientation depends on the application of fairness, reciprocity, and universal moral principles in the abstract features of ethical situations. At the pretraditional level of moral reasoning, individuals are egocentric while selecting behaviors that will help them avoid punishment and maximize self-interest (Kohlberg, 1969, 1976). By contrast, Gilligan (1982) proposed the ethics of care in response to methodological concerns about Kohlberg's(1969; 1976) research. Kohlberg examined hypothetical dilemmas among male respondents instead of the real moral dilemmas they experienced. Gilligan examined women who experienced real moral dilemmas and revealed evidence of an alternative moral orientation characterized by authentic relationships that reflect concerns about understanding the subjective experience and requirements of others, and responding to them in a genuine manner. Unlike Kohlberg, Gilligan proposed that individuals who demonstrate a care orientation do not focus on adjudicating between competing rights. According to Gilligan, individuals with a care orientation focus on identifying creative methods of simultaneously fulfilling competitive responsibilities for others (Jaffee and Shibley-Hyde, 2000).

Moral orientation refers to the manner in which an individual handles dilemma decisions. This topic is not often discussed in the context of cognitive scientific development and practice. In addition, when the stimuli related to an individual's self-concept differ (i.e., self-relevance), the individual's judgment about the orientation of justice and care also differ. Studies have demonstrated that individual factors (such as attitudes and motivations) and cultural values are two integral sources of influence (Cottone and Claus, 2000; Haidt and Joseph, 2004; Yang, 2004; Greene, 2013). For example, some studies have noted that cultural values (traditionality and modernity) provide a better reflection of individuals' performance and objectively interpret the internal motivation of their participation in social activities (Miller and Bersoff, 1992; Hong and Mallorie, 2004; Kagitcibasi, 2013; Shin et al., 2013). Traditionality (modernity) refers to a cluster of most common psychological characteristics, such as needs, attitudes, beliefs, values, temperaments and behaviors in traditional (modern) society. Therefore, when the degree of association between the target stimulus and an individual differs (i.e., self-relevance), the behavioral reactions of individuals with different cultural values may vary in justice and care moral orientation.

The modern society is presented as a multimodernity of the convergence of Eastern and Western cultures that entangles traditionality with modernity, because these basic forms of cultural values are the key psychological elements in modern society (Shin et al., 2013). Some studies have noted that examining the psychological influences of traditionality and modernity can provide a better explanation of individual behaviors and effectively interpret the internal source of our participation in modern social activities (Chan and Palley, 2005; Kagitcibasi, 2013; Frías et al., 2014). Researches have suggested that cultural values influence moral judgment. Kohlberg's theory of moral cognitive development and Gilligan's moral dualism propose that the principle of moral orientation is applicable in various social cultures (Kyte, 1996; Jaffee and Shibley-Hyde, 2000; Ellis and Shute, 2007; Tucker et al., 2014). Few scholars also believe that moral principles are culturally specific. For example, Shweder et al. (1987) noted that individuals acquired different core moral values of the society through cross-cultural studies. These moral values reflect the cultural significance of a particular group, and indicate that when they are confronted with a moral dilemma, they exhibit preference for a specific moral orientation. Miller and Bersoff (1992) examined the differences between Americans and Indians and found that Americans indicated a typical moral justice orientation, whereas Indians indicated a typical care orientation for an identical moral dilemma. Therefore, (Yang, 2004) noted that Chinese and Western culture have different origins in terms of moral principles. The fundamental difference is that both cultural systems have different assumptions about the relationship between the individual and society. Other studies have also obtained similar findings. For example, Roberts (2017) noted that both gender and cultural dilemmas play an integral role in the judgment of justice. Suh et al. (2008) used an icon paradigm to examine that the independent (Americans) vs. interdependent (Korea) self-construal process leads to different cultural behaviors. Furthermore, the results strongly suggested that differences in self-construal processes underlined cross-cultural differences in life satisfaction judgments.

Recent studies have demonstrated that examining the cultural icon prime paradigm is an effective method for studying the dynamic interaction between culture and psychology. To examine how cultural values affect the corresponding psychological processes, most studies have used experiments with higher internal validity with regards to various elements, such as cultural symbols, representations, and norms. For instance, Friedman et al. (2012) used cultural priming paradigm to reveal that managers who have been abroad switch their cultural orientation as a result of being shown Western or Chinese cultural icons, and this effect occurs when “environmental” priming is used, and also confirmed that this effect is found when examining pay allocation decisions in addition to attribution patterns. In addition, Hu and Liu (2009) recently used a classic cultural icon prime paradigm to reveal that Chinese young people showed priority to the principles of justice and care in moral dilemmas under traditional and modern cultural values.

Observations regarding the behavioral interactions in terms of moral decisions, cultural values, and self-relevant processing have triggered researchers to consider a pertinent question: does the priming of psychological modernity or traditionality substantially influence self-relevant processing on moral orientation? One of the problems that arise is that most studies have taken an explicit rather than implicit approach. Few studies in this domain have reported on the implicit level. More importantly, some studies have indicated the discrepancy between findings based on the implicit and explicit levels (Deshon and Alexander, 1996; Asendorpf et al., 2002; Schnabel et al., 2010).

Research has indicated that the IAT is a flexible and relatively straightforward tool for assessing the strengths of associations between different concepts, thus contributing notably to its widespread use in research (Nosek et al., 2007). IAT scripts are based on a seven-block (seven-task) structure; if no opposite category is evident, single-attribute IATs (SA-IATs) are deemed superior to traditional bipolar-attribute IATs (Penke et al., 2006; Seval Gündemir et al., 2014). Therefore, our research employed an SA-IAT to derive the relevant results. Thus, we aimed to use cultural icon prime paradigm to uncover the influence of self-relevance and cultural values (psychological tradition and modernity) on implicit justice and care level. Previous studies suggested that effect of self-relevance of moral orientation exists both at the implicit and explicit level (Hu and Liu, 2009, November.; Bian and Yan, 2015). More importantly, the results on the implicit level were more stable (Schneider et al., 2015; Shi et al., 2015; Burnside et al., 2018).

Based on the aforementioned studies, we sought to clarify their relationship at the implicit level by examining the following hypotheses: (1) cultural values can predict the self-reference effect on moral justice or moral care; (2) participants in the traditional culture group exhibit more differential effects on different self-referential stimuli (self, friend, or stranger) at the implicit justice level, whereas those in the modern culture group exhibit less differential effects at the implicit justice level; and (3) participants in the traditional culture group exhibit more differential effects on different self-referential stimuli (self, friend, or stranger) at the implicit care level, whereas those in the modern culture group exhibit less differential effects at the implicit care level.

## Methods

### Overview of Procedure and Design

We collected data using two experiments with similar procedures. Therefore, the procedures and results of both experiments are reported together. We used moral justice as the trait category label for an SA-IAT in Experiment 1, whereas moral care was used as the trait category label for Experiment 2. These trait categories were represented using single-attributes (e.g., “fairness” and “concern”). We informed the participants that the experiments focused on personality traits. The experiments lasted for approximately 25 min, and we promised and delivered feedback to the participants about their results after they completed the tasks. The cultural value icons and order of combined tasks within IATs were counterbalanced among participants. We designed each experiment using a 3 (cultural value icon: traditionality vs. modernity vs. no culture) × 3 (self-referential stimulus: self vs. friend vs. stranger) mixed factorial design, with repeated measures on the first factor. The dependent variables were the response time of the implicit moral justice and moral care experiments.

### Participants

A total of 126 (63 pairs of friends; 59 male participants; and 67 female participants; average age of 23.93 years; age 18–26 years) and 114 (57 pairs of friends; 54 male participants; and 60 female participants; average age of 21.56 years; age 17–26 years) healthy college students participated in Experiment 1 and 2, respectively. The participants were right-handed, had normal or corrected-to-normal vision, and had no history of neurological or psychiatric disorders. After the experiments concluded, we paid the participants ¥50. The Ethics Committee of Hunan Normal University approved this study.

### Materials

Based on the cultural icon paradigm, cultural icons were used as a stimulus (Hong et al., 2000; Hu and Liu, 2009). Similarly sized sheets of blank paper were used as the control stimulus (no culture cue; 470 pixels × 220 pixels; Hu, 2011). We asked 30 respondents to indicate their responses using a 9-point Likert scale (1 = not at all, 9 = to a great extent) to determine the extent to which cultural icons were represented.

The results indicated that the ratings of traditional cultural icons (8.23) was significantly higher than the sheets of similarly sized sheets of blank paper [1.21, *t*_(29)_ = 25.34, *p* < 0.001], and the ratings of modern cultural icons (8.16) was significantly higher than that those for similarly sized sheets of blank paper [1.32, *t*_(29)_ = −27.61, *p* < 0.001]. Therefore, we used 72 pictures (24 pictures of traditional culture, modern culture, and no culture) as the cultural cue stimuli for these Chinese participants. After reviewing recent studies (Chen et al., 2011; Fan et al., 2016), we presented self-relevant names with varying degrees of correlation as the target stimuli: (a) those with high correlations to self-material, such as the participant's name; (b) those moderately correlated with self-material, such as the name of the participant's friend; and (c) those with low correlations to self-material, such as a stranger's name. We presented stimuli from each class of self-referential stimulus.

### Procedure

#### Trial Blocks

In both studies, Single-attribute IAT (SA-IAT) used a standard seven-block procedure (Greenwald et al., 1998; Nosek et al., 2007). Target concepts were the names of self, friend and stranger, but only justice/care served as the (unipolar) attribute. The testing and analysis procedures were the same as for the justice/care IAT. Table 1 presents an example of justice SA-IAT task.

**Table 1:** Single-attribute IAT for justice: Task sequence (One example of justice SA-IAT).

			**Response key assignment**	
**Block**	**No. of trials**	**Task**	**Left key (Key F)**	**Right key (Key J)**
1	40	Target justice	Jie Deng	Yin Wang
2	40+80	Initial combined task	Jie Deng	Yin Wang, justice
3	40+80	Reversed combined task	Jie Deng, jusitice	Yin Wang

Participants started with single tasks (20 trials each) of the target concepts (“self” vs. “friend”) and the attribute concepts (“justice”/”care”). This was followed by the combined task of these concepts. Finally, the second combined task employed the reversely paired justice of target and attribute concepts.

#### Timing Details

In each trail, a fixation cross was presented for 200 ms, followed by a black screen presented for a random interval between 250 and 500 ms. A priming stimulus then were presented in white letters against the black background screen, centered in the display and remaining on screen until the subject's response. A feedback (“Right”/”Wrong”) was given and appeared for 500 ms, followed by a black screen presented for 1,000 ms before the next trials.

## Results

### Data Reduction

The data for each trial block included response latencies (in milliseconds) and error rates. The solution used for these was to recode values below 300 to 300 ms and those above 3,000 to 3,000 ms (Greenwald et al., 1998). We then log-transformed latencies in order to use a statistic that had satisfactory stability of variance for analyses (Greenwald et al., 1998). Also, the first two trials of each block were dropped because of their typically lengthened latencies. Analyses of error rates are not described in detail.

### IATs

IAT scores (i.e., the “IAT-effect”) were based on the difference in mean response latencies between the two combined blocks of different target–attribute pairings. Scores were calculated as using the d' algorithm (Greenwald et al., 1998). A positive IAT-effect is interpreted as a stronger association for the category pairing in the initial combined task—for attitude-IATs it may as well be interpreted as a preference for one concept over the other (Greenwald et al., 1998).

Scores in both studies were coded such that high scores represent high level of justice/care. Internal consistencies were estimated over separate scores for the two sub-blocks of the combined tasks (i.e., one sub-block of 40 and one sub-block of 80 trials).

Attribute stimuli were selected from Zhang et al. (2007) collection of 215 traits that had been rated for valence, arousal and social desirability. We selected 24 Words that were rated as prototypical for their respective attributes and balanced the IAT categories for social desirability and valence.

### Descriptive Statistics and Mean Differences

The descriptive statistics and internal consistencies of the main variables are depicted in Table 2. As shown in Table 2 all IATs showed satisfactory internal consistencies. IAT error rates were *M* = 7.48%, *SD* = 4.39% and *M* = 6.32%, *SD* = 5.91%, for justice and care attribute IATs in Study 1 and Study 2, respectively. No participant had error rates higher than 25%.

**Table 2:** Internal consistencies (Cronbach's α) and descriptive statistics of implicit measures.

**Variable**	**Experiment 1 (*****N*** **=** **126) (Justice SA-IAT)**			**Experiment 2 (*****N*** **=** **114) (Care SA-IAT)**		
	**α**	***M***	***SD***	**α**	***M***	***SD***
**IATs (in ms)**						
Traditionality	0.83	679.86	120.05	0.76	644.67	90.73
Modernity	0.78	516.19	109.46	0.85	506.20	105.55
No culture	0.75	542.89	119.30	0.71	538.01	95.38

### Effects of Priming Different Cultural Values on Implicit Justice Judgment

**Figure 1:**
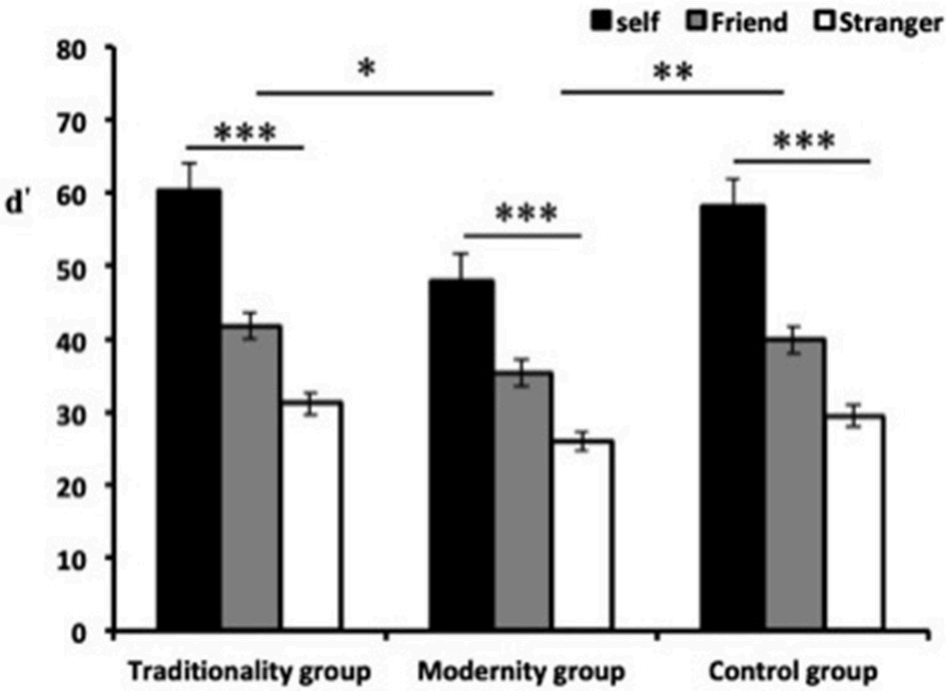
d′ values of implicit justice SA-IATs under varying cultural value conditions. ^*^p < 0.05, ^**^p < 0.01, ^***^p < 0.001.

To examine the influence of self-relevance on implicit justice judgment, we used the effect size measure “d” as the dependent variable, whereas the correlation between self-relevance and cultural values was determined using a 3 × 3 ANOVA with repeated measures on the first factor (Figure 1). The results indicated that the effect of self-relevance and cultural values were both significant, [*F*_(2, 492)_ = 228.89, *p* < 0.001, $\eta_{\text{p}}^{2}$ = 0.65], and [*F*_(2, 123)_ = 4.18, *p* < 0.05, $\eta_{\text{p}}^{2}$ = 0.36]. The *post hoc* comparisons indicate that participants' implicit justice judgments varied among the self-referential stimuli, as presented in the following differential order: self < friend < stranger, thus suggesting that the participants' justice judgments were more strict toward strangers than those for their friends and themselves. [*ts*_(125)_ = −19.28 ~ −5.05, *ps* < 0.001]. Moreover, the modernity group indicated smaller IAT effects than the control group [*t*_(82)_ = −2.22, *p* < 0.05] and the traditionality group [*t*_(82)_ = −2.90, *p* < 0.01]. However, the correlation between the control and traditionality groups was not significant (*p* > 0.05). In addition, the interaction between self-relevance and cultural values were significant [*F*_(5, 492)_ = 2.69, *p* < 0.01, η^2^_*p*_ = 0.45]. A simple effects analysis revealed that regardless of the priming conditions, the target stimulus elicited more strict justice judgments toward strangers than friends and themselves. Specifically, the target stimulus under traditional culture conditions elicited stronger justice awareness [*F*_(2, 120)_ = 52.68, *p* < 0.001] than the control group under neutral conditions [*F*_(2, 120)_ = 37.03, *p* < 0.001] and modern culture conditions [*F*_(2, 120)_ = 24.69, *p* < 0.001]. The *post hoc* multiple comparisons indicated that the highly self-relevant (self) stimulus elicited more strict justice judgments than other names, whereas moderately self-relevant names (friend) elicited more strict justice judgments than non-self-relevant names (stranger) [all *Fs*_(2, 120)_ > 21.94, all *p* < 0.001].

### Effects of Priming Different Cultural Values on Implicit Care Judgment

**Figure 2:**
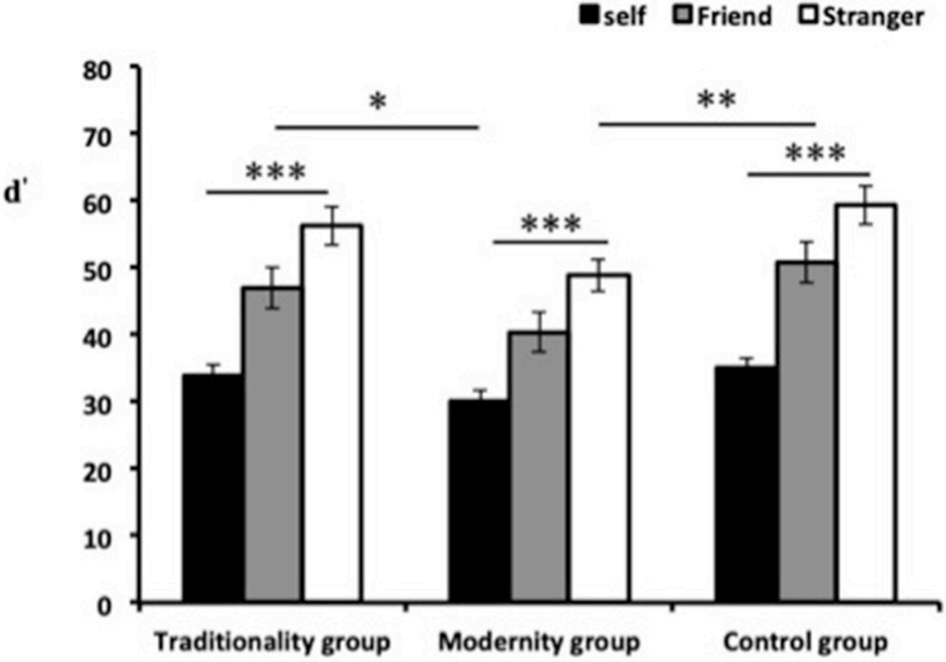
d′ values of implicit care SA-IATs under varying cultural value conditions. ^*^p < 0.05, ^**^p < 0.01, ^***^p < 0.001.

To examine the influence of self-relevance on the implicit care judgment, the effect size measure “d” was used as the dependent variable, the correlation between self-relevance and cultural values was determined using a 3 × 3 ANOVA with repeated measures on the first factor (see Figure 2). The results showed that the effect of self-relevance and cultural values were both significant, [*F*_(2, 440)_ = 234.26, *p* < 0.001, $\eta_{\text{p}}^{2}$ = 0.68], and [*F*_(2, 110)_ = 4.86, *p* < 0.05, $\eta_{\text{p}}^{2}$ = 0.28]. The *post hoc* comparisons indicated that participants' implicit care judgments varied among the self-referential stimuli, as presented in the following differential order: self > friend > stranger, thus suggesting that the participants showed less concern attitudes toward strangers than those for friends and themselves. [*ts*_(113)_ = −19.33 ~ −8.35, *ps* < 0.001]. Besides, the traditionality group indicated larger IAT effects than the modernity group [*t*_(74)_ = 3.18, *p* < 0.05], the control group indicated larger IAT effects than the modernity group [*t*_(74)_ = 2.26, *p* < 0.01]. However, the correlation between the traditionality and control groups was not significant (*p* > 0.05). In addition, the interaction between self-relevance and cultural values were significant, [*F*_(5, 440)_ = 3.05, *p* < 0.01, $\eta_{\text{p}}^{2}$ = 0.25]. A simple effects analysis revealed that regardless of the priming conditions, the target stimulus elicited less care judgments toward strangers than friends and themselves. Specifically, the target stimulus under traditional culture conditions elicited stronger care awareness [*F*_(2, 120)_ = 54.66, *p* < 0.001] than the control group under neutral condition [*F*_(2, 107)_ = 35.06, *p* < 0.001] and modern culture conditions [*F*_(2, 107)_ = 25.86, *p* < 0.001]. The *post hoc* multiple comparisons indicated that the highly self-relevant stimulus (self) elicited more care attitudes than other names, while as moderately self-relevant names (friend) elicited more concern judgments than non-self-relevant names (stranger) [all *Fs*_(2, 107)_ > 22.64, all *p* < 0.001].

## Discussion

We employed the SA-IAT and the cultural icon prime paradigm to examine whether and how self-relevance and cultural values influence implicit moral orientation (justice and care) processing. The findings showed a clear self-relevance effect: the highly self-relevant stimulus (self-names) elicited a stronger justice attitude than the names of friends and strangers in the modern cultural setting (context), whereas they elicited a weaker care attitude than the names of friends and strangers in the traditional cultural context. Moreover, the findings demonstrated variations in the justice and care orientation of the participants of the control and modernity groups, thus indicating that different cultural value orientations influence the moral justice or care attitudes of the target objects of different self-relevance at the implicit level.

The results of Experiment 1 indicate that participants' implicit justice judgments varied among the self-referential stimuli, as presented in the following differential order: ${{\text{d}}^{\prime }}_{\text{self}}{>{\text{d}}^{\prime }}_{\text{friend}}{>{\text{d}}^{\prime }}_{\text{stranger}}$. Participants spent the shortest response time to associate justice attributes with strangers, followed by those for their friends. Moreover, participants spent the longest response time to associate justice attitudes with themselves. In other words, participants strongly associated justice with the low self-relevant stimulus (names of strangers) compared with the moderate self-relevant stimulus (names of friends) and high self-relevant stimulus (their own names). This indicates that participants' sensibility to self-relevant stimuli related to justice gradually increased; that is to say, the tendency to adapt a justice attitude toward different self-relevant stimuli gradually became stricter. Moreover, the priming of different cultural values has varying effects on the differential order of justice attitude. Participants exhibited the lowest differential order in justice attitude in the context of the modernity group, whereas no significant difference was observed between the traditionality and control groups (*p* > 0.05). This result is consistent with that of explicit justice orientation (Hu and Liu, 2009; Bian and Yan, 2015). Pluralistic modern information, such as autonomy, democracy, and equality, modulates the “privateness” of justice judgments, whereas the differential order of justice was not exacerbated in the traditionality group. This may be due to the deep-rooted feudalistic ideology in China. The self-relevance effect remained significant even in the blank context (control group), indicating that no difference was evident with the traditional culture context (Chun, 1995; Tan et al., 2015).

The correlation between self-relevance and cultural values was significant, thus demonstrating that both cultural values and self-relevance influenced the implicit justice attitude. Compared with the control group, the priming of traditional cultural values aggravated the differential order of justice attitude, and the differential sequence was performed from near to far and from close to sparse, which caused differences in the justice attitude toward different self-relevant stimuli to further expand (Otten and Epstude, 2006; Le et al., 2007). Therefore, justice attitudes strengthen in response to estranged targets, thus reflecting the phenomenon of “being tolerant toward those who are close to you, but strict toward those who are unfamiliar” in the context of traditional culture. Studies have also noted that traditional traits have been internalized into people's subconscious and become automatic behaviors. The priming of traditional cultural values only affects the differential level of justice attitude and causes no other differences (Chan and Palley, 2005; Jin, 2007; Wang et al., 2016).

However, the results indicate a significant difference in the justice attitude toward self-relevant stimuli between the modern culture and control groups. The differences in the attitude tendency gradually decreased, indicating that the modernity context moderated the differential order of justice attitude. Moreover, regardless of the self-relevance level, the priming of modern cultural values can weaken the performance of the differential order and reduce the degree of “privateness.”

The results of Experiment 2 indicated that participants' implicit care judgments varied among the self-referential stimuli, as presented in the following differential order: ${{\text{d}}^{\prime }}_{\text{self}}{>{\text{d}}^{\prime }}_{\text{friend}}{>{\text{d}}^{\prime }}_{\text{stranger}}$, thus suggesting that participants spent the longest response time to associate care attributes with strangers, followed by that of their friends, whereas participants had the shortest response time to associate care attitudes with themselves. In other words, participants strongly associated care with the high self-relevant stimulus (their own names) compared with the moderate self-relevant stimulus (names of their friends) and high self-relevant stimulus (names of strangers). This indicates that participants' sensibility toward self-relevant stimulus in relation to care gradually decreases; therefore, the tendency of care attitude toward different self-relevant stimuli gradually weakens. Moreover, the priming of different cultural values has varying effects on the differential order of justice attitude. Participants exhibited the highest differential order in care attitude in the context of the modernity group, and the differential performance was more significant in the control group than in the modernity group. Therefore, participants indicated the lowest differential order in the care orientation in the modernity context. Studies have also demonstrated that individuals associate positive stimuli with high self-relevance in the traditional context, whereas they exhibit a weaker differential attitude in the modernity context (Northoff et al., 2009; Fan et al., 2013; Zhan et al., 2016).

Consistent with the aforementioned findings, we also found that the priming of traditional cultural values accelerated the differential order in care orientation, whereas this “privateness and differential order” phenomenon was weakened in the modern culture context. The reason may be that traditional cultural values activate Confucian beliefs, such as “*Qinqin*,” “*Zunzun*,” and “*Renlun*,” which have a deep-rooted influence on people's care orientation (Yan et al., 2013). By contrast, the elements of modernity reflect more democratic, equal, independent, and inclusive thoughts. Individuals may exhibit less consideration for relationships with the self-relevant stimulus in terms of care judgment for diversified characteristics in modern society context. The correlation between self-relevance and cultural values was significant, indicating that both cultural values and self-relevance influence the implicit care attitude. Compared with the control group, those who were subject to the priming of traditional cultural had an aggravated differential order of care attitude, and the differential sequence was performed from near to far and from close to sparse, which caused further expansions in the differences between care attitudes and self-relevance (Otten and Epstude, 2006; Tan et al., 2015). Participants also expressed increased concerns regarding the privacy of highly self-relevant stimulus, whereas they had less concerns regarding those of lower self-relevance.

Studies have also noted that participants responded with increased positivity toward those they had personal relationships with; whereas they responded with increased negativity toward those they were unfamiliar with (Fan et al., 2013; Tan et al., 2015; Wang et al., 2016). Our findings are consistent with these studies, and revealed that the differences in care attitude for different self-relevant stimuli were negligible between the modernity and control groups; in other words, the modernity context moderated the differential order of care attitude. Therefore, unlike other factors, the effects of the modern cultural context were reconciled, and individuals' weakened differential performance in terms of care was universal. Therefore, the modern cultural context weakened the performance of the differential order and reduces the extent of moral “privateness” regardless of the proximity or unfamiliarity of relationships between individuals and others.

## Conclusion

Taken together, in addition to the weakening effect of partial on justice and care orientation processing such as task-switching, perceptual matching reported in the previous studies, the present study further showed that culture values can weaken social cognitive processing, such as that associated with self-relevance. This self-related weakening effect occurred at both implicit justice and care orientation in the modern culture context, but not in the traditional culture context, whereas traditional culture aggravates the self-related effect in justice orientation. These findings suggest that culture values can influence self-perception in an implicit manner. Future studies should focus on specific characteristics of modern culture and self-relevant stimuli to investigate the weakening effect of moral orientation on self-processing, particularly using high-spatial-resolution fMRI to uncover neural substrates that mediate this weakening effect, and how these neural activities are related to the justice and care attitudes.

## Author Contributions

JB and LY: conceived and designed the experiments. JB, LL, QL, and JS: performed the experiments. JB, LL, XZ, and JD: analyzed the data. JB and LY: contributed reagents, materials, and analysis tools. JB and LL: wrote the paper.

### Conflict of Interest Statement

The authors declare that the research was conducted in the absence of any commercial or financial relationships that could be construed as a potential conflict of interest.

## References

[B1] Asendorpf J. Banse R. Mücke D. (2002). Double dissociation between implicit and explicit personality self-concept: the case of shy behavior. J. Pers. Soc. Psychol. 83, 380–393. 10.1037/0022-3514.83.2.380 12150235

[B2] Bian J. F. Yan L. S. (2015). The influence of interpersonal relationship between children aged 5-12 years on moral justice and moral care. Preschool Educ. Res. 38–44. 10.13861/j.cnki.sece.2015.05.006

[B3] Burnside K. Wright K. Poulin-Dubois D. (2018). Social orienting predicts implicit false belief understanding in preschoolers. J. Exp. Child Psychol. 175:167. 10.1016/j.jecp.2018.05.015 30025256

[B4] Chan L. W. Palley H. A. (2005). The use of traditional chinese culture and values in social work health care related interventions in hong kong. Health Soc. Work 30:76. 10.1093/hsw/30.1.76 15847241

[B5] Chen J. Yuan J. Feng T. Chen A. Gu B. Li H. (2011). Temporal features of the degree effect in self-relevance: neural correlates. Biol. Psychol. 87, 290–295. 10.1016/j.biopsycho.2011.03.012 21470572

[B6] Chun A. (1995). An oriental orientalism: the paradox of tradition and modernity in nationalist Taiwan. Hist. Anthropol. 9, 27–56. 10.1080/02757206.1995.9960869

[B7] Cottone R. R. Claus R. E. (2000). Ethical decision-making models: a review of the literature. J. Counsel. Dev. 78, 275–283. 10.1002/j.1556-6676.2000.tb01908.x 14626235

[B8] Deshon R. P. Alexander R. A. (1996). Goal setting effects on implicit and explicit learning of complex tasks. Organ. Behav. Hum. Dec. Process. 65, 18–36. 10.1006/obhd.1996.0002

[B9] Ellis A. A. Shute R. (2007). Teacher responses to bullying in relation to moral orientation and seriousness of bullying. Br. J. Educ. Psychol. 77, 649–663. 10.1348/000709906x163405 17908380

[B10] Fan W. Chen J. Wang X.-Y. Cai R. Tan Q. Chen Y. (2013). Electrophysiological correlation of the degree of self-reference effect. PLoS ONE 8:e80289. 10.1371/journal.pone.0080289 24312467 PMC3846566

[B11] Fan W. Zhong Y. Li J. Yang Z. Zhan Y. Cai R.. (2016). Negative emotion weakens thedegree of self-reference effect: evidence from ERPs. Front. Psychol. 7:1408. 10.3389/fpsyg.2016.01408 27733836 PMC5039173

[B12] Frías M. T. Shaver P. R. Díaz-Loving R. (2014). Individualism and collectivism as moderators of the association between attachment insecurities, coping, and social support. J. Soc. Pers. Relat. 31, 33–31. 10.1177/0265407513484631

[B13] Friedman R. Liu W. Chi S. C. S. Hong Y. Y. Sung L. K. (2012). Cross-cultural management and bicultural identity integration: when does experience abroad lead to appropriate cultural switching?. Int. J. Intercul. Relation. 36, 130–139. 10.1016/j.ijintrel.2011.03.002

[B14] Gilligan C. (1982). In a Different Voice: Psychological Theory and Women's Development. Cambridge, MA: Harvard University Press 10.1017/s0360966900023859

[B15] Greene J. (2013). Moral Tribes: Emotion, Reason, and the Gap Between Us and Them. New York, NY: The Penguin Press 10.1080/03057240.2015.1012365

[B16] Greenwald A. G. McGhee D. E. Schwartz J. L. (1998). Measuring individual differences in implicit cognition: the implicit association test. J. Pers. Soc. Psychol. 74, 1464–1480. 10.1016/s0191-8869(98)00234-7 9654756

[B17] Haidt J. Joseph C. (2004). Intuitive ethics: how innately prepared intuitions generate culturally variable virtues. Daedalus 133, 155–166. 10.1162/0011526042365555

[B18] Hong Y. Morris M. W. Chiu C. Benet-Martínez V. (2000). Multicultural minds: a dynamic constructivist approach to culture and cognition. Am. Psychol. 55, 709–720. 10.1037/0003-066X.55.7.709 10916861

[B19] Hong Y. Y. Mallorie L. A. M. (2004). A dynamic constructivist approach to culture: lessons learned from personality psychology. J. Res. Pers. 38, 59–67. 10.1016/j.jrp.2003.09.003

[B20] Hu X. M. (2011). The Influence of Psychological Tradition and Modernity on the Moral Dilemma of Young People in Chinese Changing Cultural. Unpublished Master's Dissertation, Beijing Normal University.

[B21] Hu X. M. Liu L. (2009). Research progress and future prospects of cultural psychology, in Paper Presented at the Meeting of National Psychological Academic Conference (Shandong).

[B22] Jaffee S. Shibley-Hyde J. (2000). Gender differences in moral orientation: a meta-analysis. Psychol. Bull. 126, 703–726. 10.1037//0033-2909.126.5.703 10989620

[B23] Jin G. (2007). Socialism and tradition: the formation and development of modern chinese political culture. J. Contemp. China 2, 3–17. 10.1080/10670569308724171

[B24] Kagitcibasi C. (2013). Adolescent autonomy-relatedness and the family in cultural context: what is optimal? J. Res. Adolesc. 23, 223–235. 10.1111/jora.12041

[B25] Kohlberg L. (1969). Stages in the Development of Moral Thought and Action. New York, NY: Holt, Rinehart & Winston.

[B26] Kohlberg L. (1976). Moral stages and moralization: the cognitive-developmental approach, in Moral Development and Behavior, ed Lickona T. (New York, NY: Holt, Rinehart and Winston), 31–53.

[B27] Kyte R. (1996). Moral reasoning as perception: a reading of Carol Gilligan. Hypatia 11, 97–113. 10.1111/j.1527-2001.1996.tb01017.x

[B28] Le B. Moss W. B. Mashek D. (2007). Assessing relationship closeness online moving from an interval-scaled to continuous measure of including others in the self. Soc. Sci. Comput. Rev. 25, 405–409. 10.1177/0894439307297693

[B29] Miller J. G. Bersoff D. M. (1992). Culture and moral judgment: how are conflicts between justice and interpersonal responsibilities resolved? J. Pers. Soc. Psychol. 62, 541–554. 10.1037/0022-3514.62.4.541 1583583

[B30] Northoff G. Schneider F. Rotte M. Matthiae C. Tempelmann C. Wiebking C. (2009). Differential parametric modulation of self-relatedness and emotions in different brain regions. Hum. Brain Mapp. 30, 369–382. 10.1002/hbm.20510 18064583 PMC6870760

[B31] Nosek B. A. Greenwald A. G. Banaji M. R. (2007). The implicit association test at age 7: a methodological and conceptual review, in Automatic Processes in Social Thinking and Behavior, ed Bargh J. A. (London: Psychology Press), 265–292. 10.31234/osf.io/sv8bv

[B32] Otten S. Epstude K. (2006). Overlapping mental representations of self, ingroup, and outgroup: unraveling self-stereotyping and self-anchoring. Person. Soc. Psychol. Bull. 32, 957–969. 10.1177/0146167206287254 16738028

[B33] Penke L. Eichstaedt J. Asendorpf J. B. (2006). Single-attribute implicit association tests (sa-iat) for the assessment of unipolar constructs. The case of sociosexuality. Exp. Psychol. 53:283. 10.1027/1618-3169.53.4.283 17176660

[B34] Roberts R. (2017). Gender, justice, and the problem of culture: from customary law to human rights in tanzania by dorothy l. hodgson (review). Am. Anthropol. 61, 258–260. 10.1017/asr.2018.20

[B35] Schnabel K. Asendorpf J. B. Greenwald A. G. (2010). Understanding and using the implicit association test: v. measuring semantic aspects of trait self-concepts. Eur. J. Personal. 22, 695–706. 10.1002/per.697

[B36] Schneider D. Slaughter V. P. Dux P. E. (2015). What do we know about implicit false-belief tracking? Psychon. Bull. Rev. 22, 21–12. 10.3758/s13423-014-0644-z 24847901

[B37] Seval Gündemir Homan A. C. Dreu C. K. W. D. Vugt M. V. (2014). Think leader, think white? capturing and weakening an implicit pro-white leadership bias. PLoS ONE 9:e83915. 10.1371/journal.pone.0083915 24416181 PMC3885528

[B38] Shi H. Wang X. Yi J. Zhu X. Zhang X. Yang J. (2015). Default mode network alterations during implicit emotional faces processing in first-episode, treatment-naive major depression patients. Front. Psychol. 6:1198. 10.3389/fpsyg.2015.01198 26322003 PMC4533249

[B39] Shin H. Dovidio J. F. Napier J. L. (2013). Cultural differences in targets of stigmatization between individual-and group-oriented cultures. Basic Appl. Soc. Psych. 35, 98–108. 10.1080/01973533.2012.746604

[B40] Shweder R. A. Mahapatra M. Miller J. G. (1987). Culture and Moral Development, in The Emergence of Morality in Young Children, eds Kagan J. Lamb S. (Chicago, IL: University of Chicago Press), 1–83.

[B41] Suh E. M. Diener E. Updegraff J. A. (2008). From culture to priming conditions: self-construal influences on life satisfaction judgments. J. Cross Cult. Psychol. 39, 33–15. 10.1007/978-90-481-2352-0_7

[B42] Tan Q. Zhan Y. Gao S. Fan W. Chen J. Zhong Y. (2015). Closer the relatives are, more intimate and similar we are: kinship effects on self-other overlap. Personal. Individ. Differ. 73, 77–11. 10.1016/j.paid.2014.08.038

[B43] Tucker C. M. Moradi B. Wall W. Nghiem K. (2014). Roles of perceived provider cultural sensitivity and health care justice in african american/black patients' satisfaction with provider. J. Clin. Psychol. Med. Settings 21:282. 10.1007/s10880-014-9397-0 24913783

[B44] Wang X. Tu M. Yang R. Guo J. Yuan Z. Liu W. (2016). Determinants of pro-environmental consumption intention in rural china: the role of traditional cultures, personal attitudes and reference groups. Asian J. Soc. Psychol. 19, 215–224. 10.1111/ajsp.12142

[B45] Yan L. S. Zhou L. Zeng L. (2013). Differences in moral justice and care: on collective bias in Chinese moral orientation. Psychol. Sci. 1168–1175. 10.16719/j.cnki.1671-6981.2013.05.011

[B46] Yang G. S. (2004). Theoretical analysis and experimental research on chinese self: social orientation and personal orientation. J. Indig. Psychol. 2004, 2011–2080. 10.3389/fpsyg.2017.01106

[B47] Zhan Y. Jie C. Xiao X. Jin L. Yang Z. Wei F. (2016). Reward promotes self-face processing: an event-related potential study. Front. Psychol. 7:735. 10.3389/fpsyg.2016.00735 27242637 PMC4871870

[B48] Zhang D. Wang D. Yang Y. Teng F. (2007). Do personality traits predict work values of chinese college students? Soc. Behav. Personal. Int. J. 35, 1281–1294. 10.2224/sbp.2007.35.9.1281

